# Congenital intrahepatic portosystemic shunt in 27 children: an experience and treatment strategy of a single centre in China

**DOI:** 10.3389/fped.2024.1428270

**Published:** 2024-11-28

**Authors:** Jin-Shan Zhang

**Affiliations:** Department of General Surgery, Capital Institute of Pediatrics, Beijing, China

**Keywords:** congenital intrahepatic portosystemic shunt, children, surgery, treatment, strategy

## Abstract

**Objective:**

To evaluate treatment strategies for congenital intrahepatic portosystemic shunt (CIPSS) based on the experience of treating 27 children.

**Methods:**

Between August 2017 and January 2024, our team treated 27 children with CIPSS. Twelve patients underwent surgical ligation of the portosystemic shunt, while 15 patients diagnosed prenatally received conservative treatment without surgery. All patients were followed up after diagnosis or surgery. During follow-up, blood ammonia and biochemistry tests, along with ultrasound examinations, were conducted. Clinical presentations were recorded.

**Results:**

The prenatal diagnosis rate for CIPSS using ultrasound was 74.1% (20/27). Hyperammonemia was the most common clinical manifestation, occurring in 81.5% (22/27) of cases. Jaundice and abnormal liver function were the next most frequent presentations in patients with prenatal diagnosis, with incidences of 80% (16/20) and 65% (13/20), respectively. In 12 patients undergoing surgical ligation, blood ammonia levels returned to normal, the abnormal shunt disappeared as confirmed by ultrasound and CT, and no patients developed portal vein thrombosis or portal hypertension postoperatively. In 15 patients receiving conservative treatment, 53.3% (8/15) experienced spontaneous closure of the abnormal shunt within 1–7 months (median: 3 months). Jaundice and hyperammonemia were completely resolved within 1–8 months in patients receiving conservative treatment.

**Conclusion:**

CIPSS is a curable congenital anomaly. Prenatal ultrasound is effective for detection. Conservative treatment is recommended until the age of one, followed by surgical ligation or interventional treatment for patients with persistent shunts after 1 year.

## 1 Introduction

Congenital portosystemic shunt (CPSS) is a rare vascular malformation with an incidence of 1 in 30,000 to 1 in 50,000. These abnormal shunts typically connect the portal venous system (intrahepatic portal vein, main portal vein, splenic vein, superior mesenteric vein, or inferior mesenteric vein) to the systemic circulation system (inferior vena cava, hepatic vein, iliac vein, or renal vein). Based on their location, CPSSs are classified as extrahepatic or intrahepatic. Congenital extrahepatic portosystemic shunt (CEPSS), also known as Abernethy malformation, was first described by John Abernethy in 1793. In contrast, congenital intrahepatic portosystemic shunt (CIPSS) involves abnormal shunts between the intrahepatic portal vein branches and the hepatic veins, and is less common than CEPSS. A key difference between CEPSS and CIPSS is their prognosis. Shunts in CEPSS do not typically close spontaneously, while spontaneous closure is possible in the early stages of CIPSS (1–5). This study summarizes the diagnosis and treatment experience of 27 children with CIPSS, providing insights into effective treatment strategies for this condition.

## 2 Materials and methods

Between August 2017 and January 2024, our team treated 27 children with CIPSS. From August 2017 to August 2022, 12 cases (age at operation: 13 days to 15.3 years, median: 0.5 years, male: 6, female: 6) underwent surgical ligation of the portosystemic shunt. Among these 12 cases, 5 were diagnosed through prenatal ultrasound. The remaining seven cases were diagnosed based on abdominal ultrasound findings and clinical symptoms, as detailed in Table 1. All 12 cases required surgical closure of the CIPSS due to persistent portosystemic shunting (in patients over one year of age) or severe clinical symptoms. From December 2020 to January 2024, 15 children (recent age during follow-up: 6–30 months, median: 16 months, male: 12, female: 3) with CIPSS were diagnosed prenatally and received conservative treatment without surgery. These patients have been under our care since the initial prenatal diagnosis.

**Table 1:** The general information and surgical results of 12 cases with surgical ligation.

No.	Operative age (year)	Duration of follow-up (months)	Gender	Origin of bypass vein	Clinical presentations	Surgical methods	AMM (umol/L)		Caliber (mm)	Time of diagnosis (Age, years)
							Pre	Post		
1	8.7	9	F	RPV	Hemangiomas, hyperammonemia	LRPV	52.16	36	6.2–8.8	8
2	15.3	14	F	DV	Cardiac dilatation, PH	LDV	37.55	33.03	14.8	11
3	0.4	19	F	RPV	Hemangiomas, hyperammonemia	LRPV	40.36	38.73	3.9	0.4
4	1.4	24	F	DV	Jaundice, abnormal liver function, liver tumor, hyperammonemia	LDV	52.09	20.66	8.6	1.2
5	0.2	25	M	LPV	Jaundice, abnormal liver function	LLPV	25	39.7	2.9	Prenatal
6	0.1	36	M	LPV	Hyperammonemia	LLPV	217	20.2	2–2.7	Prenatal
7	8.6	37	M	DV	Cardiac dilatation, hyperammonemia	LDV	67.44	22.97	4.6	8.3
8	21 days	43	F	LPV	Jaundice, abnormal liver function, hyperammonemia	LLPV	95	33.2	1.3, 0.8	Prenatal
9	0.6	44	M	LPV	Abnormal liver function, hyperammonemia	LLPV	47.84	38	2.8	0.6
10	6.1	48	F	RPV	Hypoxemia, PAVF	pLRPV	27.52	26.52	5.2	5
11	13 days	59	M	LPV	Jaundice, hyperammonemia	LLPV	106.9	27.2	8.4	Prenatal
12	0.1	69	M	LPV	Hyperammonemia	LLPV	81.8	31.71	4	Prenatal

LLPV, ligation of left portal vein; LRPV, ligation of right portal vein; LDV, ligation of ductus venosus; pLRPV, partial ligation of right portal vein; RPV, right portal vein; LPV, left portal vein; DV, ductus venosus; PAVF, pulmonary arteriovenous fistula; PH, pulmonary hypertension; F, Female; M, Male; Caliber, caliber of abnormal shunt measured by ultrasound; pre, pre-operative; post, post-operative.

Clinical presentations, including jaundice, abnormal liver function, and hyperammonemia, were evaluated by measuring serum levels of total bilirubin (TBIL), direct bilirubin (DBIL), indirect bilirubin (IBIL), alanine aminotransferase (ALT), aspartate transferase (AST), *γ*-glutamyl transpeptidase (GGT), and ammonia (AMM). Normal ranges for these markers may vary depending on the reagent kits used. The jaundice was evaluated by the levels of TBIL, DBIL, and IBIL. The abnormal liver function was evaluated by the levels of ALT, AST, and GGT. The hyperammonemia was evaluated by the level of AMM. A diagnosis of jaundice, abnormal liver function, or hyperammonemia was made when measured levels exceeded the established normal ranges. These normal ranges in our hospital are as follows: TBIL (3.4–20.0 µmol/L), DBIL (0–3.4 umol/L), IBIL (3.4–16.6 umol/L), ALT (7–30 U/L), AST (14–44 U/L), GGT (5–19 U/L), and AMM (11–40 µmol/L).

### 2.1 Surgical method

**Figure 1:**
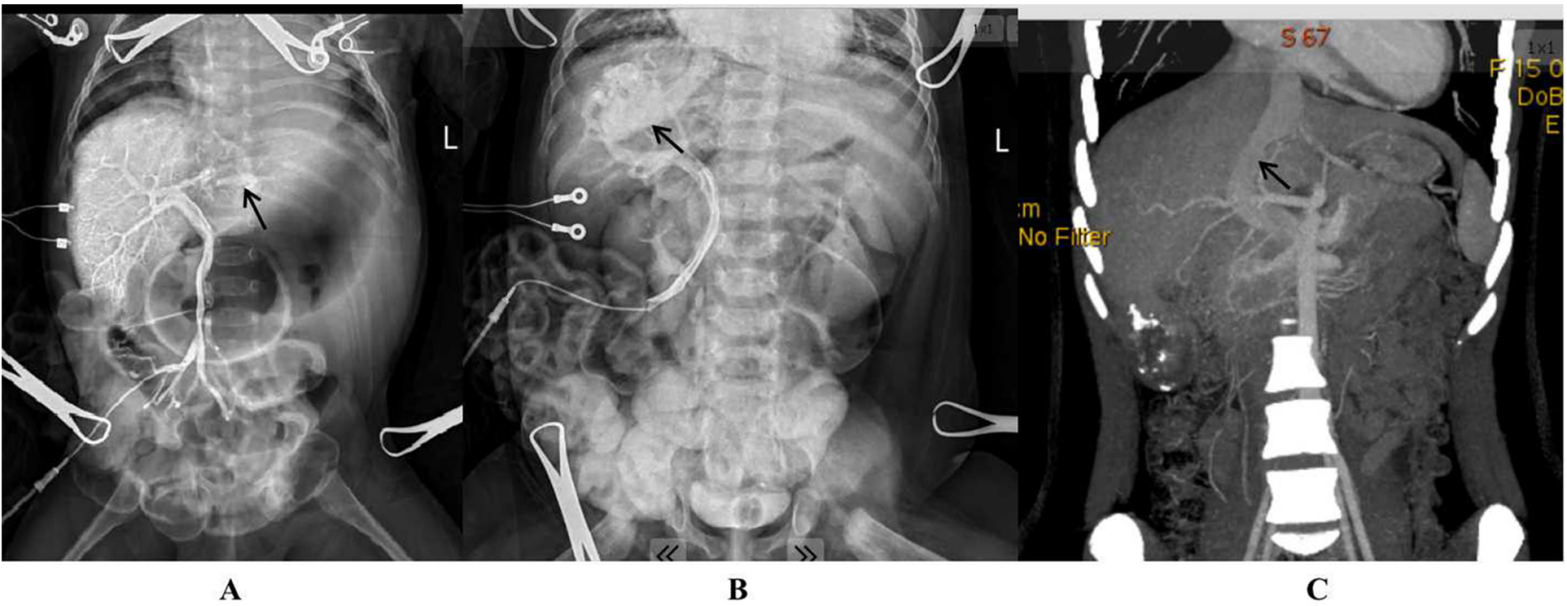
Intrahepatic portosystemic shunt (arrow) as demonstrated by intraoperative portal venography (**A**: a shunt from the left portal vein to the left hepatic vein; **B**: a shunt from the right portal vein to the right hepatic vein; **C**: a patent ductus venosus shown by preoperative CT three-dimensional imaging).

Initially, a 16G venous catheter was inserted into a branch of the superior mesenteric vein to measure portal pressure and perform portal venography. This procedure allowed for the diagnosis of CIPSS and identification of the abnormal shunt's location (Figure 1). A decision to perform complete or partial ligation of the shunt was based on portal pressure measurements after temporarily clamping the shunt. If the small intestine appeared uncongested and the occluded portal pressure did not exceed 26 cmH_2_O, complete ligation was performed. However, if portal hypertension (portal pressure >26 cmH_2_O) or significant small intestine congestion was observed during temporary occlusion, partial ligation was performed until portal pressure was reduced to 26 cmH_2_O or lower. A second operation for definitive shunt closure was scheduled 3–6 months later (6).

Postoperatively, intravenous heparin was administered to prevent portal vein thrombosis for seven days. Subsequently, oral Plavix or aspirin was used for six months to maintain anticoagulation. Follow-up visits were scheduled for 1, 3, 6 months, and then every six months thereafter. During follow-up, ultrasound and CT scans were used to evaluate the abnormal shunt, portal vein thrombosis, and spleen size. Routine blood tests, including blood biochemistry, coagulation function, and ammonia levels, were performed. Clinical presentations were also recorded.

### 2.2 Conservative method

Postnatal care included conservative management, which consisted of medical treatment, dietary adjustments, and regular monitoring.

Medical management aimed to alleviate and resolve clinical presentations, including abnormal liver function, jaundice, and hyperammonemia. The treatment regimen included:

(1) **Compound glycyrrhizic acid glycoside tablets:** An oral Chinese herbal medicine containing glycyrrhizin, monoammonium glycyrrhizinate, aminoacetic acid, and methionine, used to treat elevated transaminases. Glycyrrhizin has been shown to have multiple therapeutic effects on liver disease, including anti-steatosis, anti-oxidative stress, anti-inflammation, immunoregulation, anti-fibrosis, anti-cancer, and drug-drug interaction properties (7). The efficacy of Compound glycyrrhizin injections for the treatment of drug-induced liver injury was analyzed by comparing the levels of ALT and AST between the compound glycyrrhizin group and the control group (8). Results showed that the compound glycyrrhizin group had a significantly higher overall normalization rate of ALT and AST than the control group, indicating the effectiveness of compound glycyrrhizin injections in reducing ALT and AST levels in patients with drug-induced liver injury. Additionally, glycyrrhizic acid, a component of Compound glycyrrhizin, has been shown to attenuate liver fibrosis and hepatic stellate cell activation in animal models, suggesting its potential as a candidate compound for preventing or relieving liver fibrosis (9).
(2) **Ursodeoxycholic acid capsules and yinzhihuang granules:** Oral medications used to treat jaundice. Yinzhihuang granules, a traditional Chinese herbal medicine containing extracts of Artemisia capillaris Thunb., Gardenia jasminoides Ellis, Lonicera japonica Thunb., and Scutellaria baicalensis Georgi, have been shown to be effective and safe for neonatal jaundice (10).
(3) **Lactulose:** An oral medication used to treat hyperammonemia by trapping ammonia in the gut and promoting the formation of NH_4_^+^ from NH_3_. For severe hyperammonemia, intravenous arginine may be administered.

Dietary management focused on reducing blood ammonia levels. For infants over six months old, decreasing the intake of protein-rich foods, such as breast milk or powdered milk, can be beneficial.

Regular monitoring included ultrasound examinations to identify the malformation and blood tests (biochemistry and ammonia) to assess liver function, total bilirubin, and blood ammonia levels. Blood ammonia was monitored closely, with measurements performed every two weeks during the first three months, monthly from three to six months, and semi-annually thereafter until normal levels were achieved. Liver function and total bilirubin were monitored monthly until normal levels were reached. Ultrasound examinations were conducted at 1, 3, 6, and 12 months after birth, followed by every six months until spontaneous closure of the CIPSS.

### 2.3 Ethical statement

All procedures performed in studies involving human participants were under the ethical standards of the institutional research committee, the 1964 Helsinki declaration and its later amendments or comparable ethical standards. This work has been approved by the ethical committee of the Capital Institute of Pediatrics. All patients and their parents gave their informed consent before their inclusion in the study.

## 3 Results

The prenatal diagnosis rate of CIPSS by ultrasound was 74.1% (20/27). Among patients who underwent surgical ligation, the prenatal diagnosis rate was 41.7% (5/12), while it was 100% (15/15) in those receiving conservative treatment.

Hyperammonemia was the most common clinical presentation, occurring in 81.5% (22/27) of cases. Following hyperammonemia, jaundice and abnormal liver function were the next most frequent presentations in patients with prenatal diagnosis, with incidences of 80% (16/20) and 65% (13/20), respectively. Cardiac dilatation, liver tumor, hypoxemia, pulmonary arterial hypertension, and pulmonary arteriovenous fistula were observed in five patients over one year of age and were alleviated but not completely resolved after surgical ligation.

Twelve patients who underwent surgical ligation were followed for 9–69 months (median: 32.5 months). Postoperatively, their blood ammonia levels returned to normal, and the abnormal shunt was no longer visible on ultrasound or CT. There were no postoperative complications, including portal vein thrombosis or portal hypertension (Table 1).

Fifteen patients who received conservative treatment were followed for 6–30 months after birth (median: 16 months). Of these, eight experienced spontaneous closure of the abnormal shunt, as confirmed by ultrasound. The overall spontaneous closure rate was 53.3% (8/15), occurring within 1–7 months (median: 3 months) in the eight patients with spontaneous closure (Table 2). Among the five cases diagnosed after 12 months of age that did not close spontaneously, one had a persistent abnormal shunt successfully occluded through an interventional procedure at 20 months (Case No. 7 in Table 2). The remaining four cases with persistent shunts were not treated with interventional closure despite our recommendations. To date, these four untreated cases have not developed severe complications, such as cardiac dilatation, liver tumor, hypoxemia, pulmonary arterial hypertension, or pulmonary arteriovenous fistula.

**Table 2:** The information and results of 15 cases with conservative therapy.

No.	Recent age (months)	Gender	Origin of bypass vein	Clinical presentations	Age in a normal (months)				TBIL (umol/L)		ALT (U/L)		AST (U/L)		GGT (U/L)		AMM (umol/L)		Caliber (mm)	
					TBIL	ALT, AST, GGT	AMM	Spontaneous closure	After birth	Recent	After birth	Recent	After birth	Recent	After birth	Recent	After birth	Recent	After birth	Recent
1	30	M	LPV	JA, AL, HA	3	1	3	3	167.5	5	29.3	12	48	24.4	56	6	76	20.15	3.2	Closure
2	28	M	LPV	JA, AL, HA	3	3	3	2	251.3	4.6	158.2	22.6	158.1	38.6	156	13	45.37	34	1.9, 2.6^a^	Closure
3	29	M	LPV	JA, AL, HA	8	8	8	3	87	5.1	79.1	27.7	74.6	33	157	18	41	38	1	Closure
4	16	M	LPV	JA, AL, HA	8	1	1		233.9	8.2	4	25.8	52	32	188	16.5	89	14	3.9	1.6
5	26	M	RPV	HA			7	7	5.7	6.7	24.6	23.2	36.6	32.5	12	11	45.8	12.46	2.2	Closure
6	15	M	LPV	JA, AL, HA	1	2	1	1	152.5	10.2	12	10.7	23.3	24.9	188.9	15	92.7	32.44	2.8	Closure
7	20	M	DV	JA, AL	5				70.3	16.9	88.9	41.8	102.1	82.6	264	13	25.56	20.63	3.3	3.8
8	22	M	RPV	JA, AL, HA	4	7	8		316.28	7.5	13	29	33	22	334	13	91.5	24.5	3	5.3
9	18	M	RPV	AL		6			19	8.7	79	28	98	40	102	14	53^b^	62.9^b^	8.9	5.2 × 3.1^c^
10	12	F	LPV	JA, HA	4		4		223.2	4.28	34	26	33	36	32	13	60	23.4	3.4	1.9
11	6	M	LPV	JA, HA	6		6		234.1	13	19	28	35	33	118	15	42	20	2.2	1.9
12	10	M	LPV	JA, HA	4		4	4	121	10.6	11.1	22.4	30.5	45.4	105.5	23.4	73.4	25.7	1.9	Closure
13	12	M	LPV	JA, AL, HA	3	1	3	1	82.7	9.3	4.2	21.3	45	40	136	18	82.4	33.7	2	Closure
14	10	F	LPV, DV	JA, AL, HA	6	6	6	6	328.6	3.7	9	26	54	38	82	10.9	65^b^	50.9^b^	2, 2.9^a^	Closure
15	6	F	LPV, RPV	JA, AL, HA	2		6		25.8	15.68	91.2	63.3	92.9	75.3	70	17.6	91.61	22	14.8*12^c^	6*6^c^

F, female; M, male; LPV, left portal vein; RPV, right portal vein; DV, ductus venosus; a, two branches of bypass vein; b, Normal range is 16–60 umol/L; c, angiomatous change; Caliber, caliber of abnormal shunt shown by ultrasound; TBIL, total bilirubin; ALT, alanine aminotransferase; AST, aspartate transferase; AMM, ammonia; JA, Jaundice; AL, abnormal liver function; HA, hyperammonemia; the normal range of AMM is 11–40 umol/L in our hospital.

In patients receiving conservative treatment, jaundice, abnormal liver function, and hyperammonemia were common clinical presentations, with incidences of 86.7% (13/15), 73.3% (11/15), and 86.7% (13/15), respectively. The remission rate for jaundice was 100% (13/13) within 1–8 months (median: 4 months), and the elevated levels of direct bilirubin (DBIL) returned to normal in all 13 patients with jaundice (Table 3). The remission rate for liver dysfunction was 81.8% (9/11), with transaminase levels returning to normal within 1–8 months (median: 3 months). Of the 15 patients receiving conservative treatment, 13 with hyperammonemia experienced a complete remission, as evidenced by normalized blood ammonia levels (Table 2). This resulted in a 100% remission rate for hyperammonemia, with blood ammonia levels returning to normal within 1–8 months (median: 4 months).

**Table 3:** The changes of TBIL, DBIL and IBIL in 15 cases with conservative therapy.

NO.	TBIL (umol/L)		DBIL (umol/L)		IBIL (umol/L)	
	After birth	Recent	After birth	Recent	After birth	Recent
1	167.5	5	14.4	1.0	153.1	4
2	251.3	4.6	16.2	1.7	235.1	2.9
3	87	5.1	38.8	1.2	48.2	3.9
4	233.9	8.2	14.6	1.2	219.3	7
5	5.7	6.7	2.1	2.2	3.6	4.5
6	152.5	10.2	17.9	2.3	134.6	7.9
7	70.3	16.9	49.7	6.5	20.6	10.4
8	316.28	7.5	6.12	3.4	310.16	4.5
9	19	8.7	5.9	2.9	13.1	5.8
10	223.2	4.28	45.6	1.58	177.6	2.7
11	234.1	13	8.8	3	225.3	10
12	121	10.6	36.1	1.7	84.9	8.9
13	82.7	9.3	16.7	3.4	66	6.3
14	328.6	3.7	10.8	1.4	317.8	2.3
15	25.8	15.68	5.7	3.12	20.1	12.56

TBIL, total bilirubin; DBIL, direct bilirubin (conjugated bilirubin); IBIL, Indirect bilirubin (unconjugated bilirubin); the normal range of TBIL is 3.4–20.0 umol/L; the normal range of DBIL is no more than 3.4 umol/L; the normal range of IBIL is less than 16.6 umol/L.

## 4 Discussion

### 4.1 Prenatal diagnosis

CIPSS is often difficult to diagnose without specialized examinations. Approximately 27% of CIPSS cases are incidentally detected during abdominal or liver examinations for other conditions (11). Ultrasound is a crucial diagnostic tool, particularly during the prenatal period. Up to 42% of congenital portosystemic shunts (including CEPSS and CIPSS) are diagnosed through prenatal ultrasound, and this rate continues to rise (12–14). In our study, 74.1% (12/27) of CIPSS cases were first identified by prenatal ultrasound. Early diagnosis of CIPSS through prenatal ultrasound is advantageous for timely treatment. In our surgical group, some patients without prenatal diagnosis experienced severe complications, such as cardiac dilatation, liver tumors, and hypoxemia, due to delayed diagnosis and treatment. Therefore, early diagnosis of CIPSS enables prompt treatment and regular monitoring, helping to prevent complications associated with delayed care.

### 4.2 Clinical presentations

Hyperammonemia is a common clinical manifestation of CIPSS, occurring in approximately 79% of patients with congenital portosystemic shunt (CPSS) (1). Among CIPSS cases diagnosed prenatally, hyperammonemia is the most prevalent presentation, with an incidence of up to 85% (17/20). Jaundice and liver dysfunction are also common, occurring in 80% and 65% of cases, respectively.

Hyperammonemia, a risk factor for hepatic encephalopathy, is often considered an indication for surgical intervention in CIPSS (6). Early treatment is generally recommended (1). However, the question remains whether hyperammonemia alone justifies surgery in patients younger than one year old. In our study, all patients treated conservatively experienced normalization of blood ammonia levels within 1–8 months, suggesting that hyperammonemia can be effectively managed without surgical intervention in this age group. Elevated ammonia levels are a common consequence of portal-systemic shunting, as ammonia absorbed from the intestine bypass the liver. While elevated ammonia levels generally indicate the presence of a portal-systemic shunt, other factors such as dietary regime and intestinal function can also influence ammonia levels. In addition to closing the abnormal shunt, treating hyperammonemia involves dietary adjustments to limit protein intake and reduce ammonia production and absorption in the intestine. If dietary management and oral lactulose are ineffective, intravenous arginine may be considered to lower blood ammonia levels and prevent encephalopathy.

Cholestasis caused by CIPSS in neonates is often curable (15). In our study, all patients treated conservatively experienced resolution of jaundice and abnormal liver function, further supporting the notion that cholestasis alone may not necessitate surgical intervention in CIPSS (16). However, it's important to differentiate cholestasis caused by CIPSS from that resulting from biliary malformations (such as biliary atresia, biliary dysplasia, and choledochocyst), which can be identified through ultrasound, CT, or cholangiography and may coexist with CIPSS (1).

Following conservative therapy, the remission rates for jaundice, liver dysfunction, and hyperammonemia were 100% (13/13), 81.8% (9/11), and 100% (13/13), respectively. Among the patients without clinical presentations after conservative therapy, eight had spontaneous shunt closure, while others did not. This suggests that both shunt closure and conservative therapy contributed to the resolution of clinical symptoms. However, this study's limitation is the lack of direct comparison between patients who received conservative therapy and those who did not. Such a comparison would provide more definitive evidence regarding the effectiveness of conservative treatment.

### 4.3 Treatment

The primary goal of CPSS treatment is to prevent or reverse complications, including hepatic encephalopathy, liver tumor and injury, hepatopulmonary syndrome, pulmonary hypertension, and heart diseases (17, 18).

#### 4.3.1 Spontaneous closure

Spontaneous closure rates for CIPSS are generally higher than for CEPSS (47% vs. 4%), especially in patients younger than two years old (19, 20). In our study, 53.3% (8/15) of patients receiving conservative therapy experienced spontaneous CIPSS closure between 1 and 7 months, with a median age of 3 months. However, the mechanism and predictive factors for spontaneous closure remain unclear (20). Therefore, careful monitoring is recommended until the age of one, followed by reduced follow-up frequency (11).

There is no consensus regarding the optimal timing for surgical closure of congenital portosystemic shunts (CPSS) (1, 15). Some experts suggest a medical approach for patients older than two years old, based on the possibility of spontaneous closure within this age range (16). In our study, eight patients with CIPSS experienced spontaneous closure between 1 and 7 months of age. No spontaneous closures were observed after 7 months. This suggests that the likelihood of spontaneous closure significantly decreases after one year of age, and delaying surgical intervention may increase the risk of complications. In patients undergoing surgical ligation, five experienced serious complications, including cardiac dilatation, liver tumor, hypoxemia, pulmonary arterial hypertension, and pulmonary arteriovenous fistula, likely due to delayed closure.

#### 4.3.2 Surgical closure

##### 4.3.2.1 Methods of surgical closure

For patients with CIPSS that does not close spontaneously and is diagnosed after the age of one, surgical closure is generally recommended, either through surgical ligation or interventional embolization (21). Interventional embolization is often the preferred approach, but surgical ligation may be necessary for shunts with a larger diameter or shorter length. Additionally, surgical ligation may be required if complete closure cannot be achieved in a single surgical procedure, necessitating a staged approach (6). In our study, 12 children underwent surgical ligation instead of interventional embolization. The decision to prioritize surgical ligation was influenced by several factors. Firstly, our institution did not have specialists trained in interventional embolization until 2023. Secondly, interventional embolization carries certain risks, such as ectopic embolism, portal vein thrombosis, and the inability to perform staged surgery (16).

##### 4.3.2.2 Experimental occlusion before closure

Before definitive closure of CIPSS, an experimental occlusion of the abnormal shunt, using either a balloon in interventional treatment or a suture in surgical ligation, is a crucial procedure to evaluate the plasticity of the intrahepatic portal vein. Failure to perform this procedure effectively can increase the risk of portal hypertension, portal vein thrombosis, and even death following CIPSS closure.

Portal pressure is a key indicator during experimental occlusion. Previous studies involving 70 children reported the results of radiologic or surgical shunt occlusion, including portal pressure measurements in 59 and angiography in 30. These studies found that occlusion portal pressures were below 20 mm Hg in 23 children, between 20 and 29 in 20 children, and 30 mm Hg or higher (range 30–45) in 16 (1). However, a definitive cutoff value for determining whether a one- or two-stage closure is necessary remains elusive.

Previously, an occlusion portal pressure above 32 mm Hg was considered an indicator of a two-stage procedure, especially if the portal vein was not visible on the occlusion angiogram (1, 16). Recent literature suggests that a single-stage closure may be feasible if the test occlusion results in a portal pressure gradient less than 10 mm Hg and the absolute occluded portal pressure remains below 25 mm Hg (22). Therefore, a portal pressure gradient less than 10 mm Hg and an absolute occluded portal pressure below 25 mm Hg were considered indicators of a single-stage interventional closure. If these criteria are not met, surgical ligation is recommended.

In surgical ligation, an experimental occlusion must be performed again. If the portal pressure is no more than 20 mm Hg, complete ligation of the abnormal shunt can be considered. Otherwise, partial ligation may be necessary to maintain portal pressure at 20 mm Hg, followed by a staged procedure after 3–6 months (6, 16).

### 4.4 Post-operative complications and others

In addition to the anesthesia risks associated with pulmonary hypertension, the primary complications following closure of a portosystemic shunt include mesenteric or portal vein thrombosis (15), thrombus displacement, and secondary portal hypertension (17). Unlike portosystemic shunts caused by portal venous obstruction or liver cirrhosis, CPSS typically does not exhibit portal hypertension (5). Therefore, portal hypertension after CPSS closure may be attributed to vascular obstruction within the portal vein system, such as portal vein dysplasia, or potentially to liver fibrosis. Preoperative liver biopsy and experimental occlusion are essential to evaluate these factors before closing the shunt (15). Postoperative anticoagulation therapy with heparin is recommended to prevent portal vein thrombosis. In this study, all children undergoing surgical ligation received heparin, and no cases of portal vein thrombosis were observed.

The decision regarding preventive treatment for CPSS remains controversial (11). When the benefits of shunt closure outweigh the risks, preventive treatment is justified to protect patients from life-threatening complications, such as pulmonary hypertension. However, predicting the onset of complications is challenging. Therefore, it is recommended to close the shunt in children without spontaneous closure after the age of one to mitigate the risk of complications. Additionally, the intrahepatic portal vein is generally more plastic in younger patients compared to older patients, supporting the rationale for early shunt closure. In 2023, McLin VA et al. (21) proposed their recommendations regarding the timing of CIPSS closure: ① Follow all asymptomatic intrahepatic CPSS detected at birth longitudinally until spontaneous closure and 1 year beyond documented closure. ② Close asymptomatic intrahepatic CPSS if they do not close spontaneously within the first 2 years of life. ③ Close all symptomatic CPSS beyond the neonatal period.

### 4.5 Limitations

This study has several limitations. First, it is a descriptive study based on the experience of a single center. Second, the conclusion regarding the optimal timing for closure at one year of age is based on observational data and may require further confirmation. Third, the study lacks a formal statistical analysis of the outcomes. Future studies with larger sample sizes are needed to evaluate the effectiveness of conservative therapy and compare the outcomes between patients with spontaneous closure and those with open shunts undergoing conservative treatment.

## 5 Conclusions

**Figure 2:**
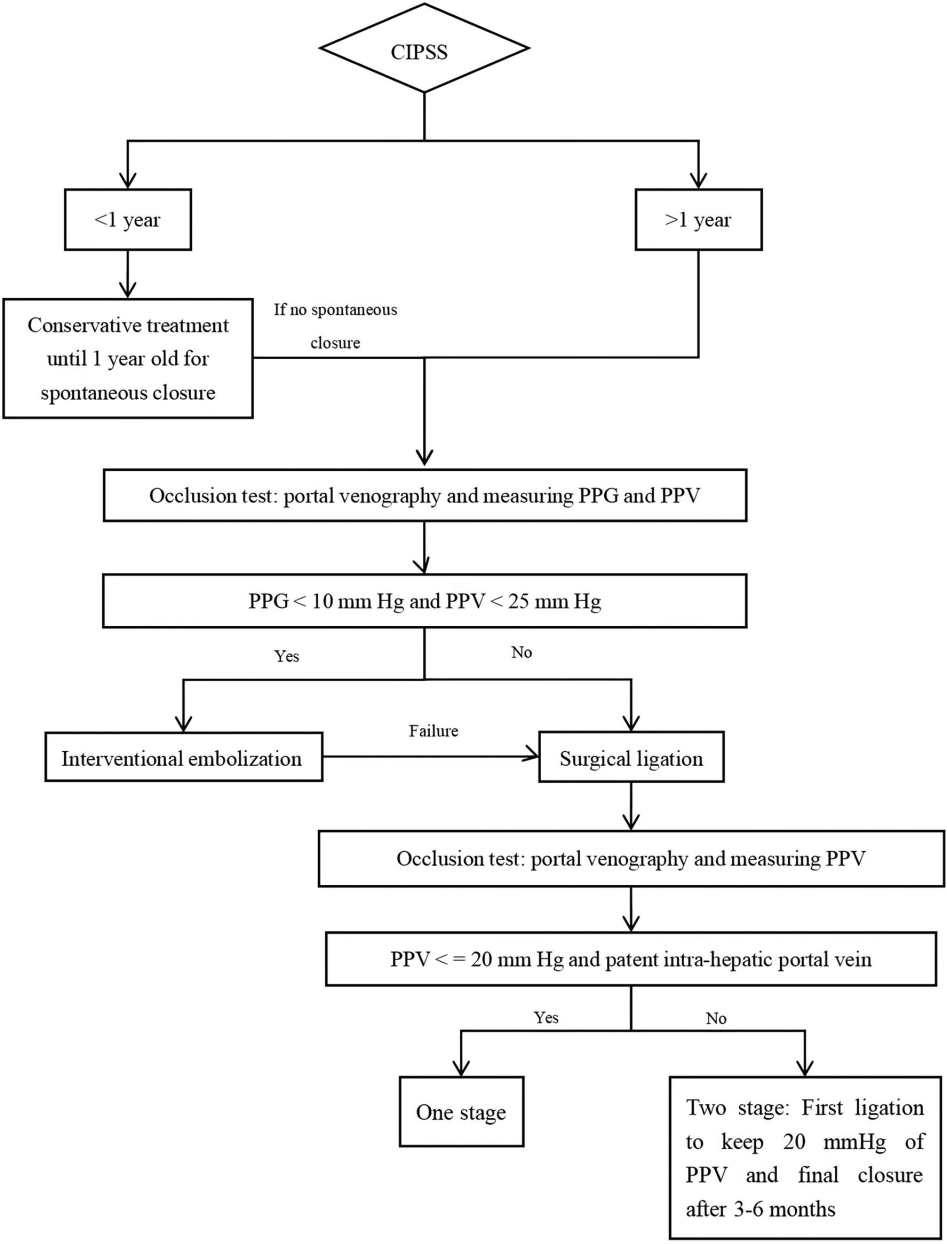
The suggested treatment strategy of CIPSS (PPG: portal pressure gradient; PPV: pressure of portal vein).

CIPSS is a curable congenital anomaly. Prenatal ultrasound is an effective diagnostic tool. We recommend the following treatment strategies: conservative treatment until the age of one, followed by surgical ligation or interventional treatment for patients with persistent shunts after one year (Figure 2).

## Data Availability

The raw data supporting the conclusions of this article will be made available by the authors, without undue reservation.
